# Supplementary material for: Clustering Electrophysiological Predisposition to Binge Drinking: An Unsupervised Machine Learning Analysis
Source: Brain Behav. 2024 Nov 22;14(11):e70157. doi: 10.1002/brb3.70157 (PMC11583822; doi:10.1002/brb3.70157)
Supplement: Supplementary file 1 — Figure S1. Distribution of alcohol consumption (Standard Alcohol Units, SAUs) throughout the population, represented in a histogram. In the X axis, the value of SAUs in the population are represented. In the Y axis, the number of participants in each group is represented. [file BRB3-14-e70157-s004.docx]

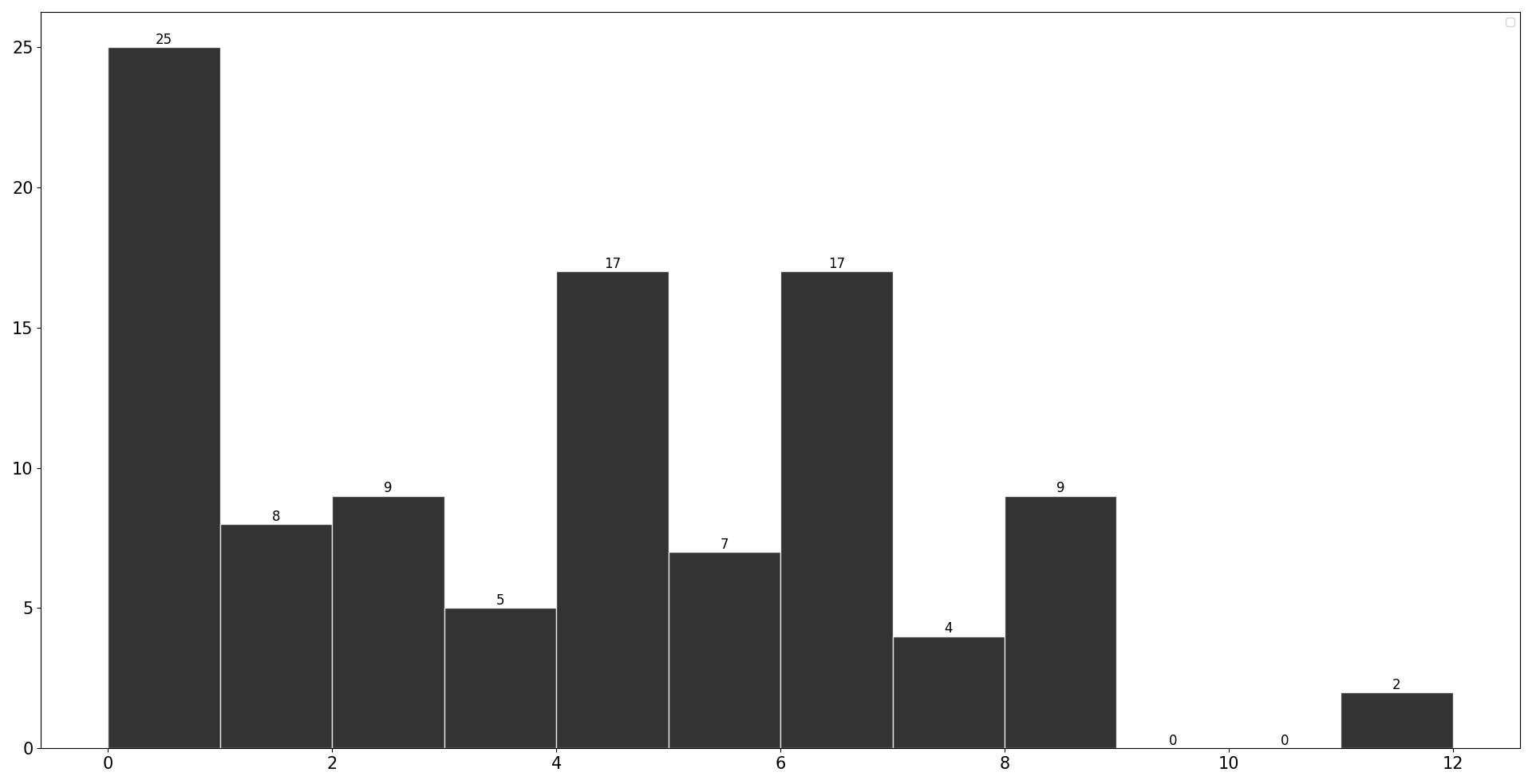


Number of Participants

Value of Alcohol Consumption (Standard Alcohol Unit values)

**Supplementary Figure 1:** distribution of alcohol consumption (Standard Alcohol Units, SAUs) throughout the population, represented in a histogram. In the X axis are represented the value of SAUs in the population. In the Y axis is represented the number of participants in each group.
