# Supplementary material for: Clustering Electrophysiological Predisposition to Binge Drinking: An Unsupervised Machine Learning Analysis
Source: Brain Behav. 2024 Nov 22;14(11):e70157. doi: 10.1002/brb3.70157 (PMC11583822; doi:10.1002/brb3.70157)
Supplement: Supplementary file 2 — Figure S2. Graphs of correlations, broken down for all areas of the AAL atlas, between electrophysiological variables (power spectrum and strength) and consumption (SAUs) for the four frequency bands of interest (A: alpha, B: beta, C: gamma, and D: theta). For each, on the left are the correlation graphs for Cluster 1, with: power‐UBEs graph (in blue), strength‐UBEs graph (in green), and the graph showing areas of overlap between both variables. On the right, the correlation graphs for Cluster 2, with: power‐UBEs graph (in blue), strength‐UBEs graph (in green), and the graph showing areas of overlap between both variables. For each of the graphs, beneath the title, the mean rho is displayed, along with its standard deviation. [file BRB3-14-e70157-s001.docx]

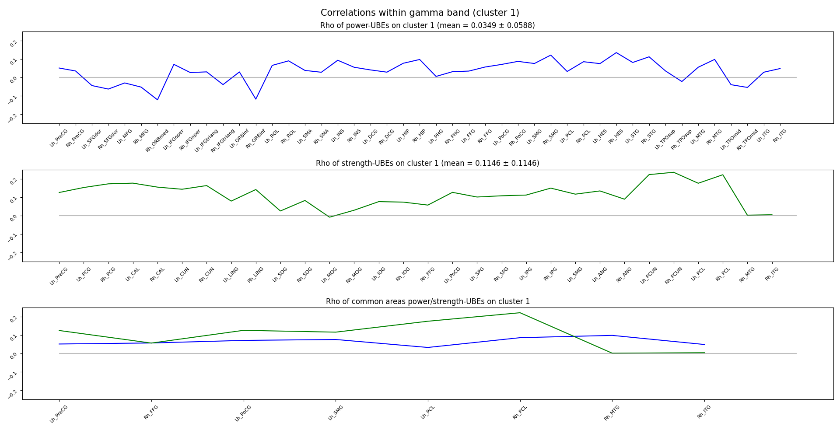

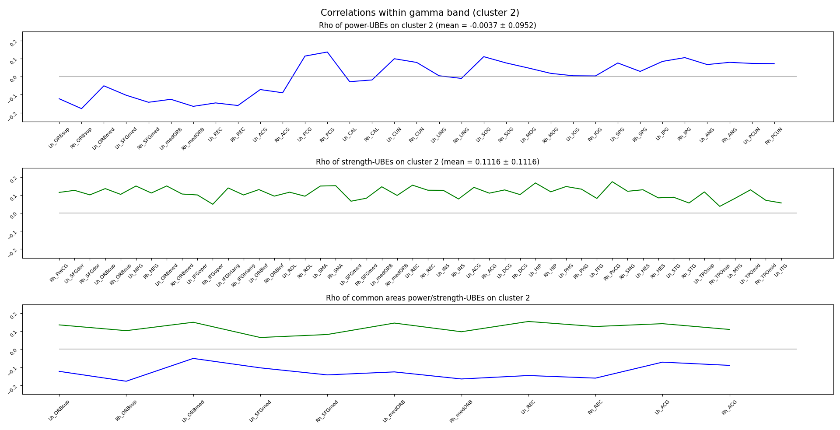

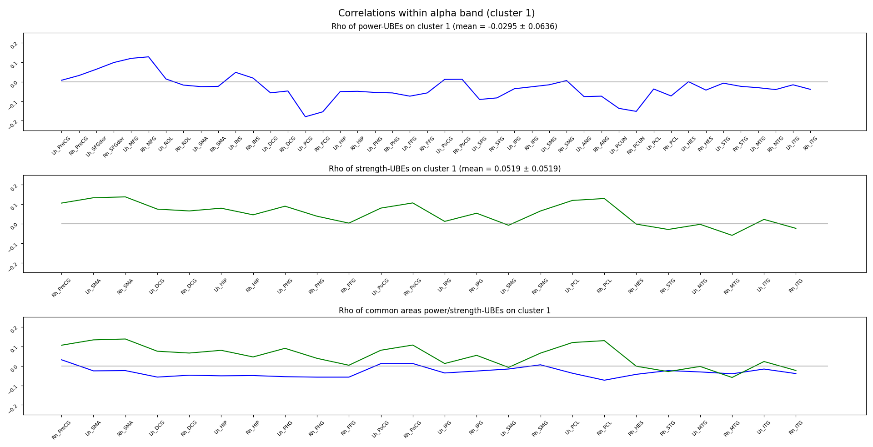

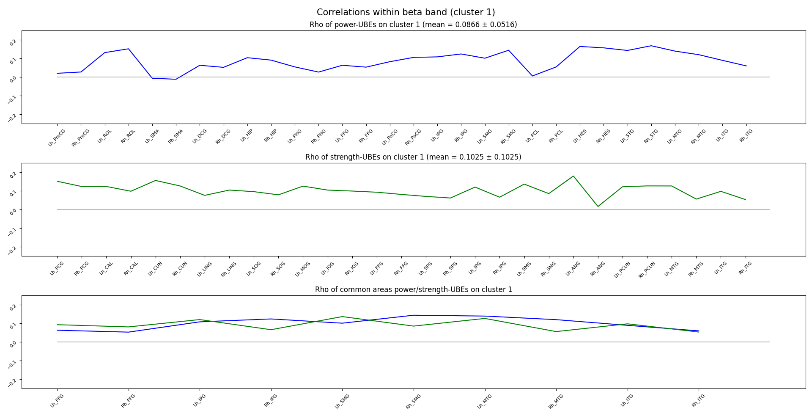

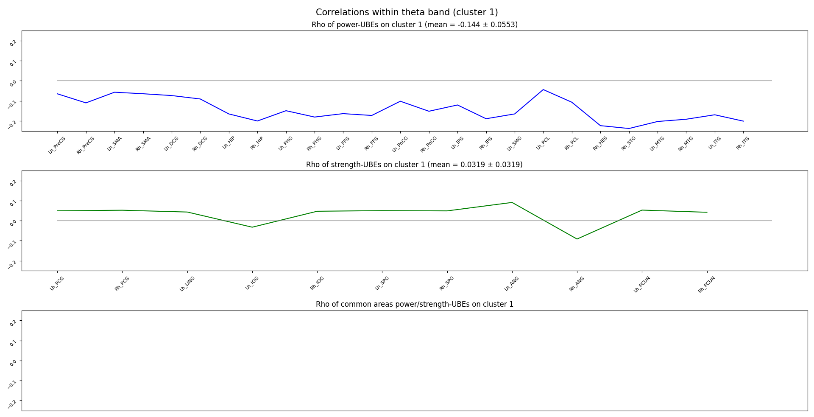

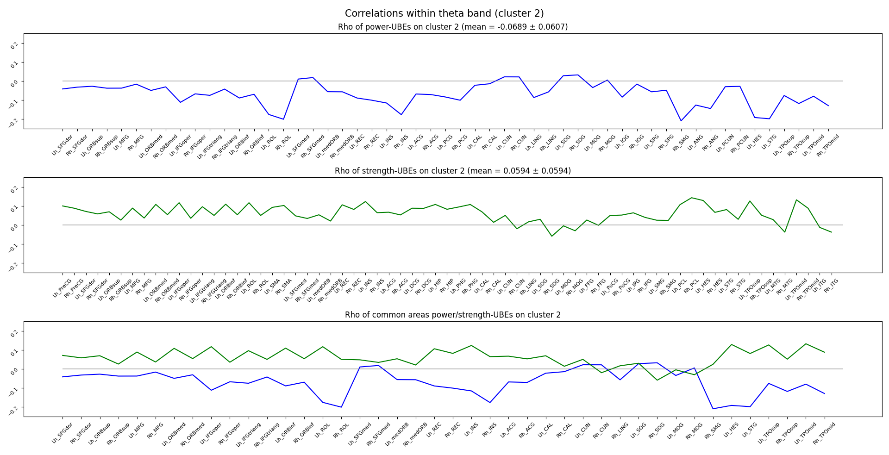

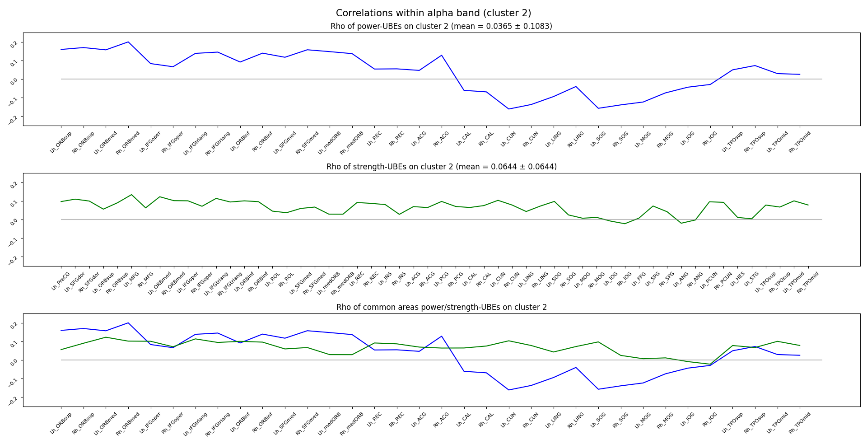

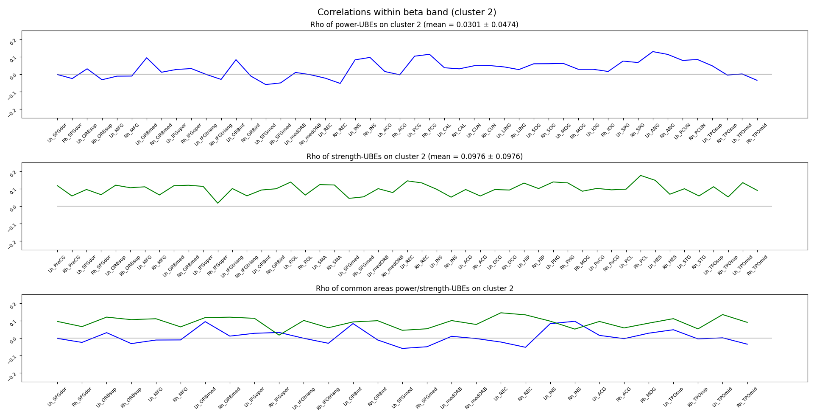


**Supplementary Figure 2:** Graphs of correlations, broken down for all areas of the AAL atlas, between electrophysiological variables (power spectrum and strength) and consumption (SAUs) for the four frequency bands of interest (**A**: alpha, **B**: beta, **C**: gamma, **D**: theta). For each, on the left are the correlation graphs for cluster 1, with: power-UBEs graph (in blue), strength-UBEs graph (in green), and the graph showing areas of overlap between both variables. On the right, the correlation graphs for cluster 2, with: power-UBEs graph (in blue), strength-UBEs graph (in green), and the graph showing areas of overlap between both variables. For each of the graphs, beneath the title, the mean *rho* is displayed, along with its standard deviation.

D)

C)

A)

B)
