# Supplementary material for: Clustering Electrophysiological Predisposition to Binge Drinking: An Unsupervised Machine Learning Analysis
Source: Brain Behav. 2024 Nov 22;14(11):e70157. doi: 10.1002/brb3.70157 (PMC11583822; doi:10.1002/brb3.70157)
Supplement: Supplementary file 3 — Figure S3. Visualization of clusterization of random data compared to visualization of real electrophysiological data (theta, alpha, beta, and gamma band, respectively). (A) Visualization of data from Cluster 1. (B) Visualization of data from Cluster 2. (C) Dendrogram and cohesion/coherence parameters of the random data clustering. In blue, the parameters of the first cluster; in red, the parameters of the second cluster. [file BRB3-14-e70157-s002.docx]

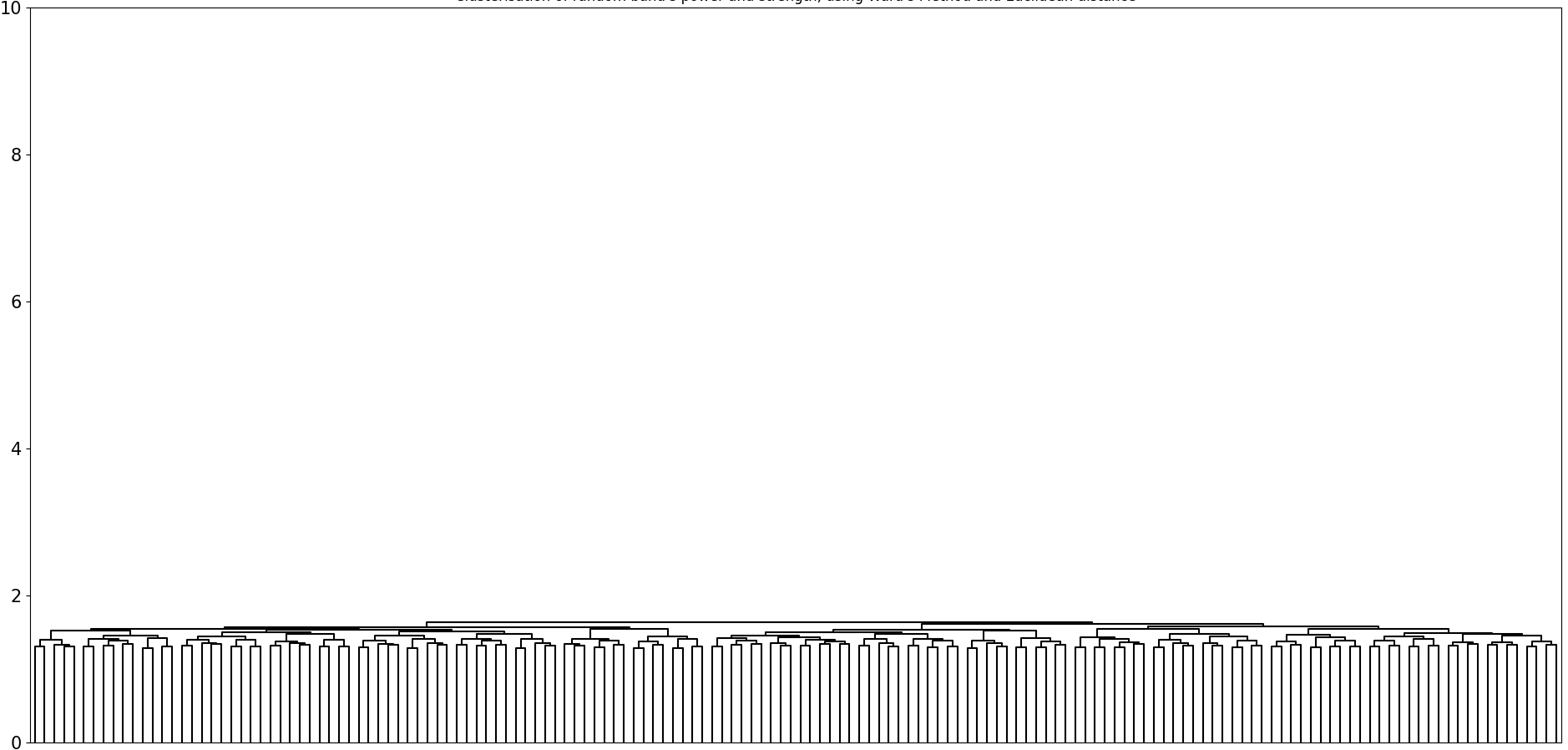

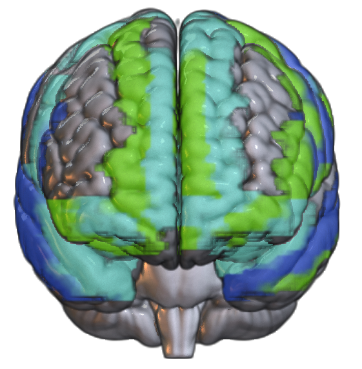

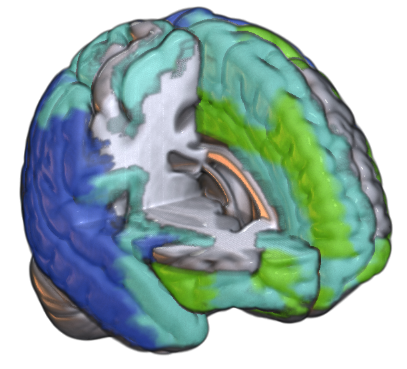

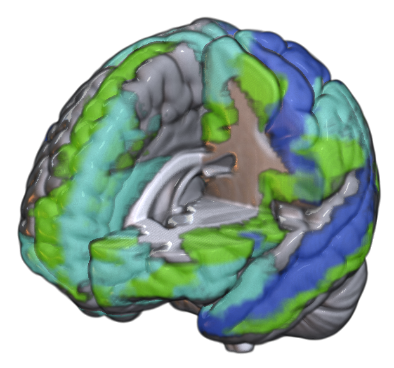

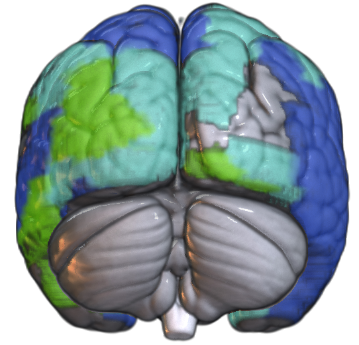

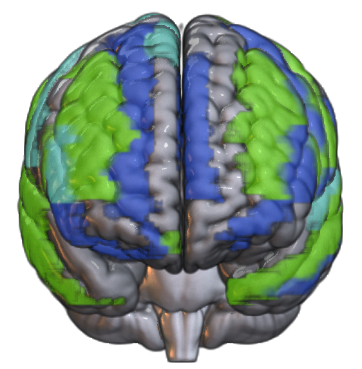

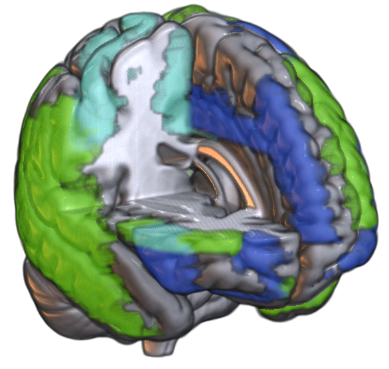

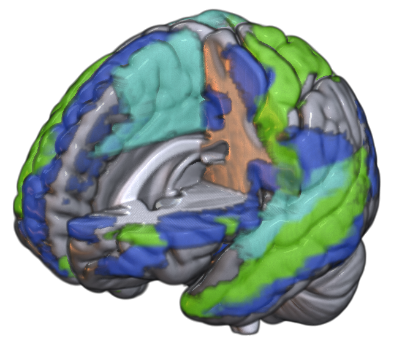

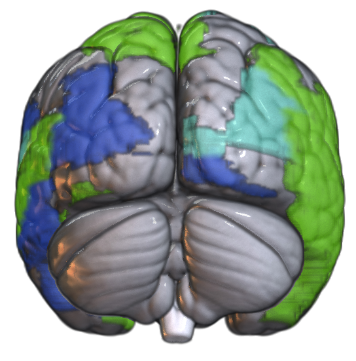

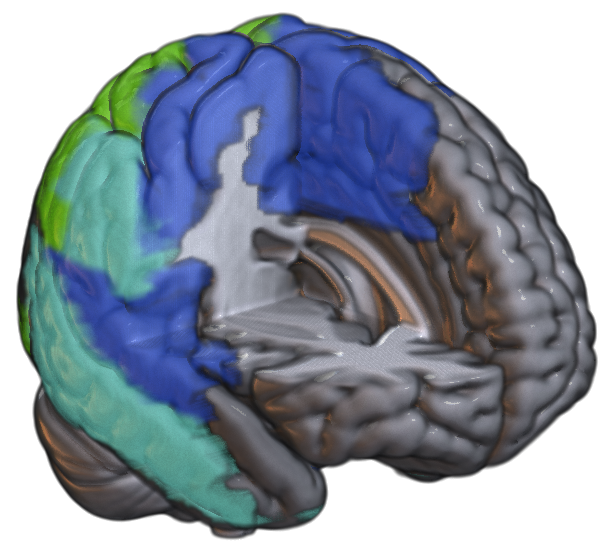

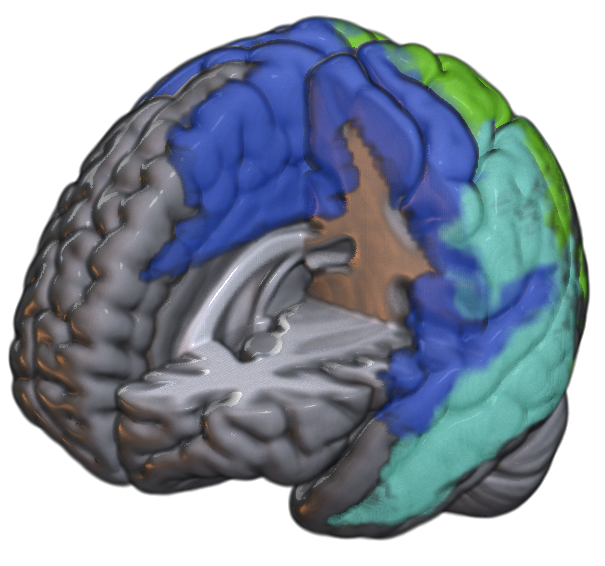

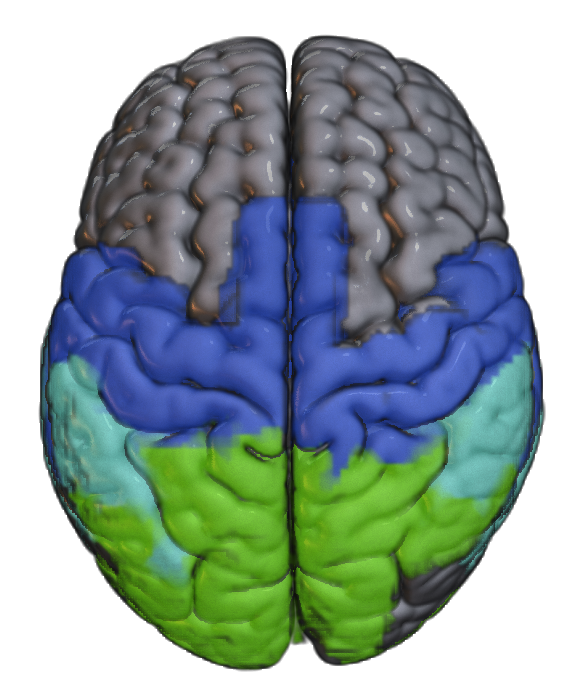

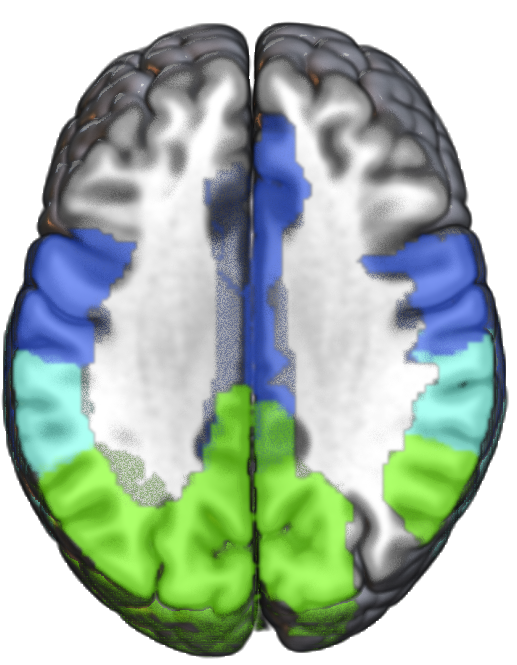

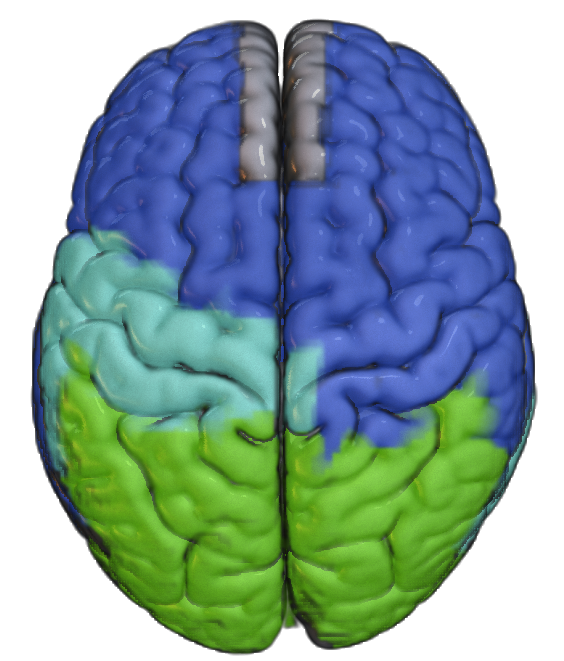

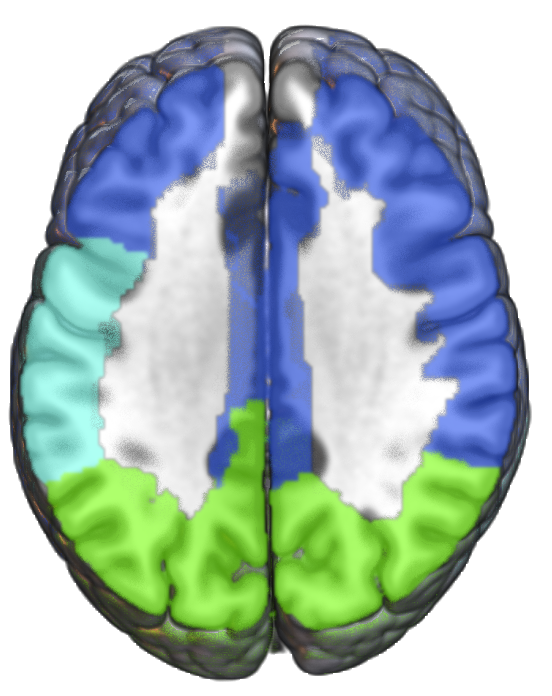

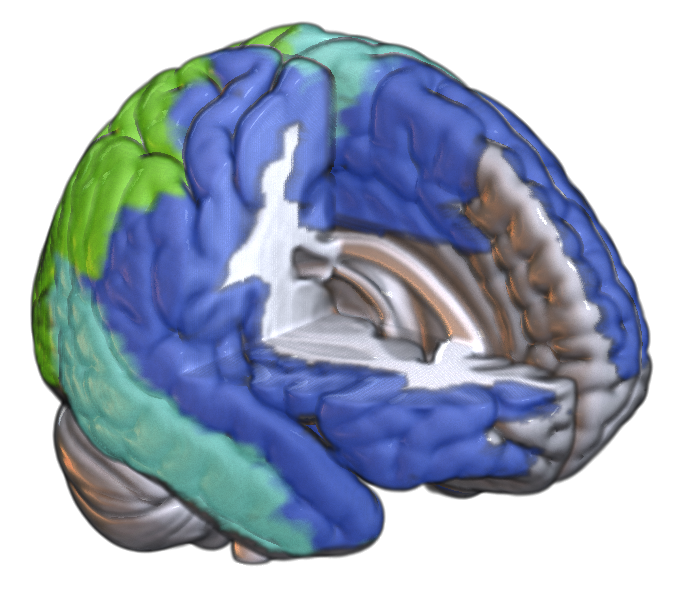

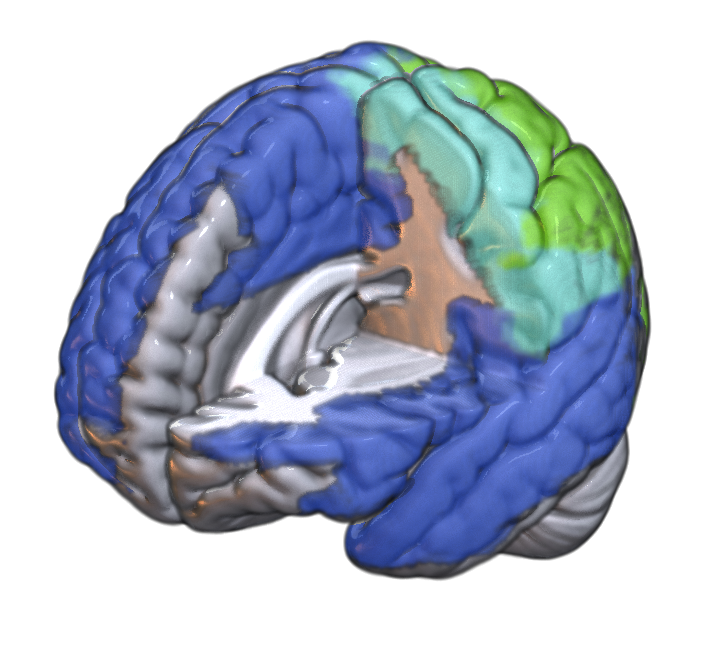

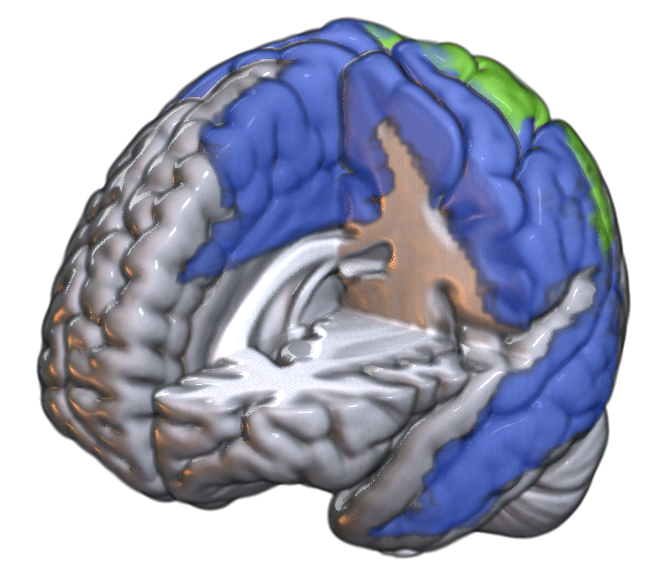

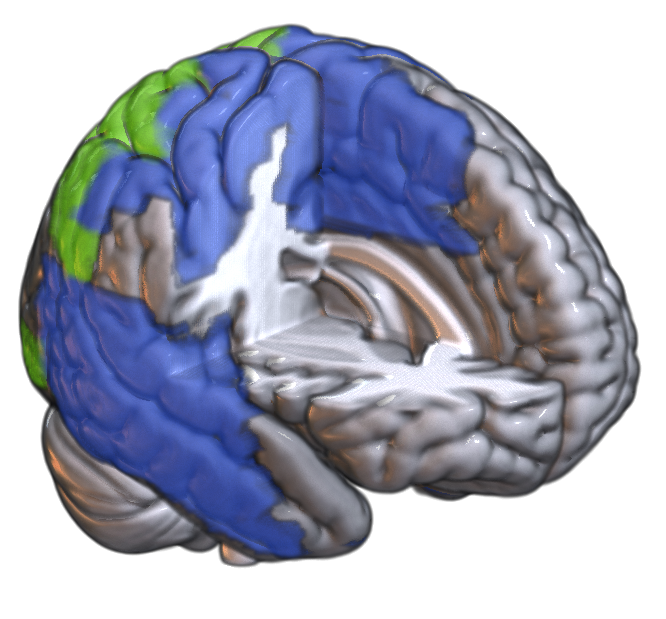

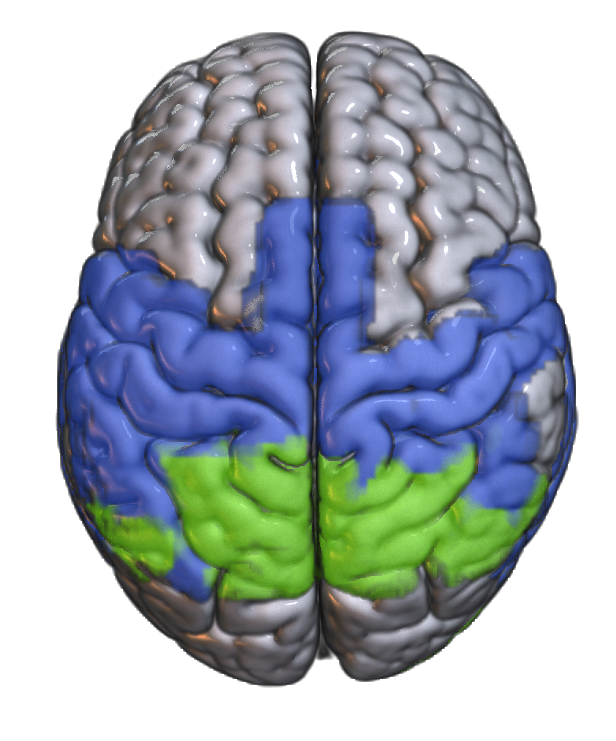

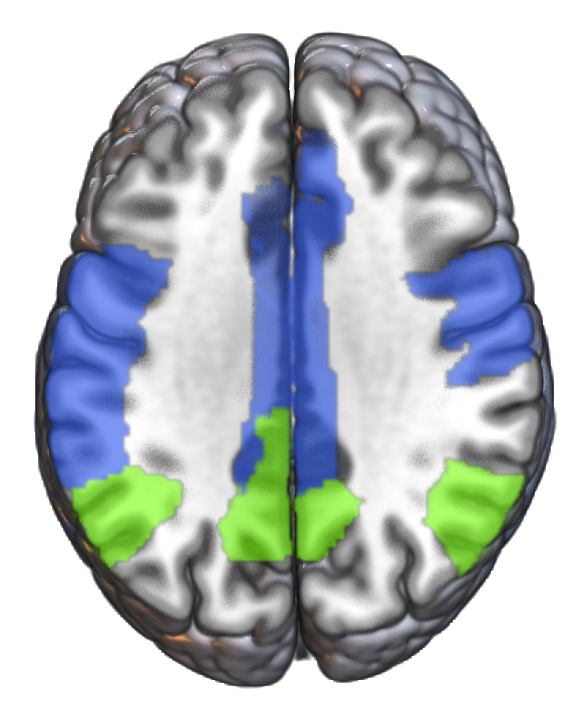

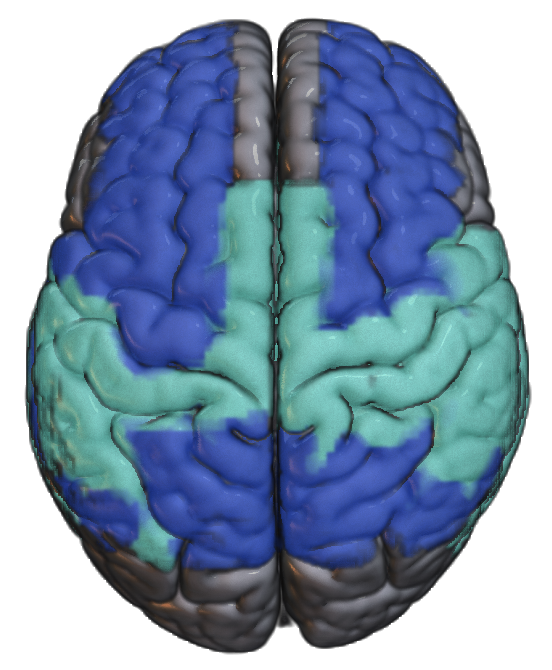

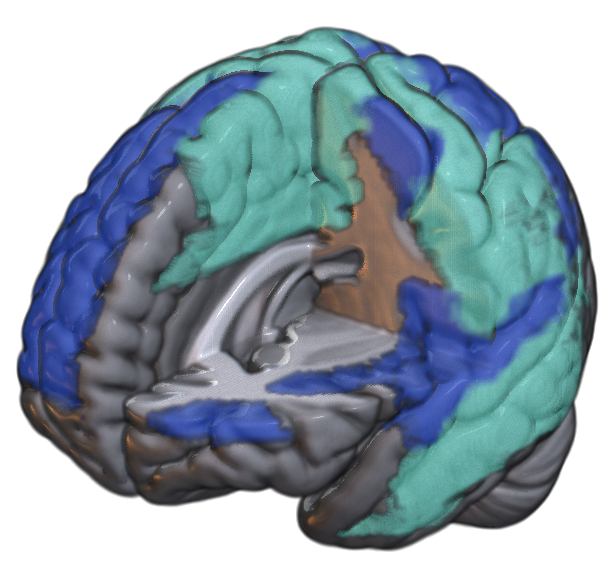

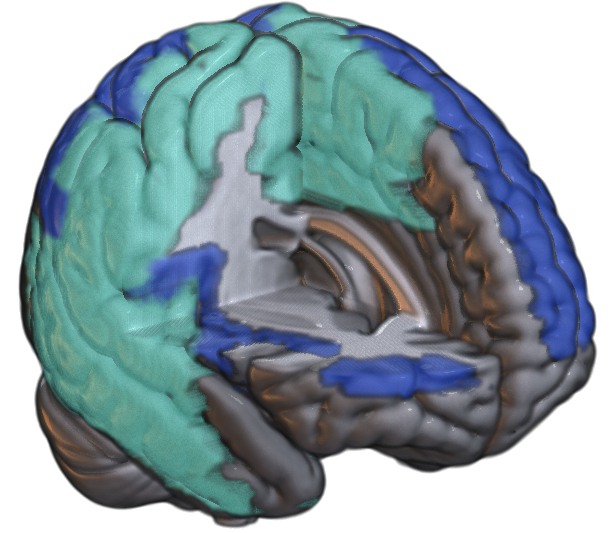

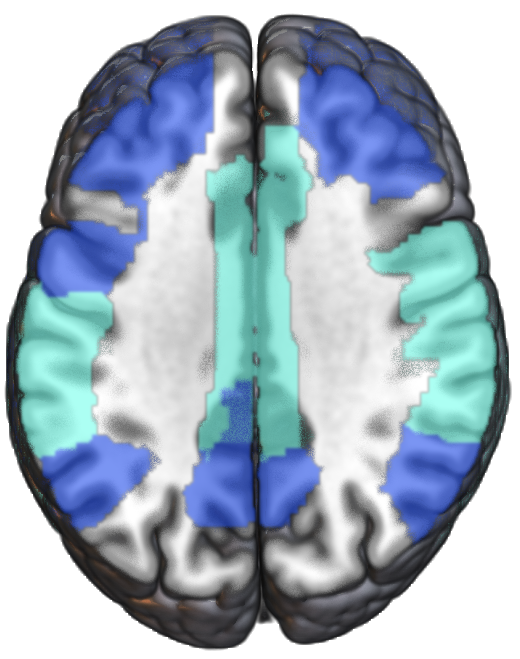

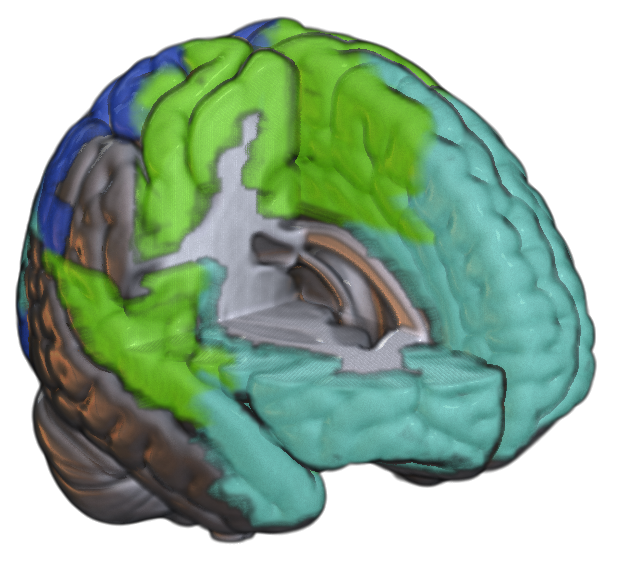

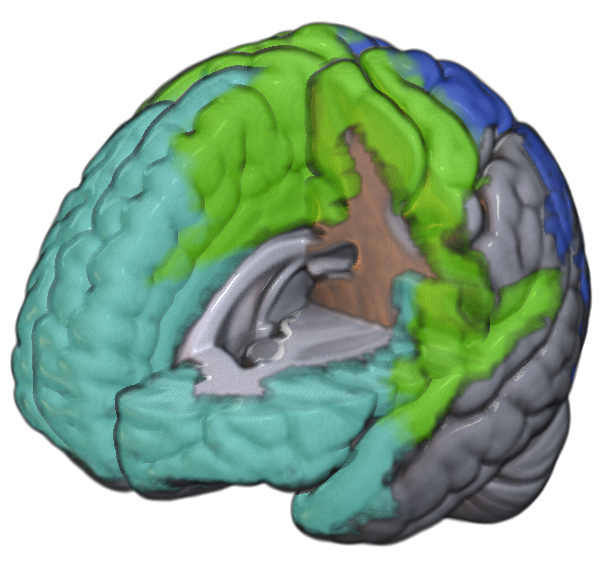

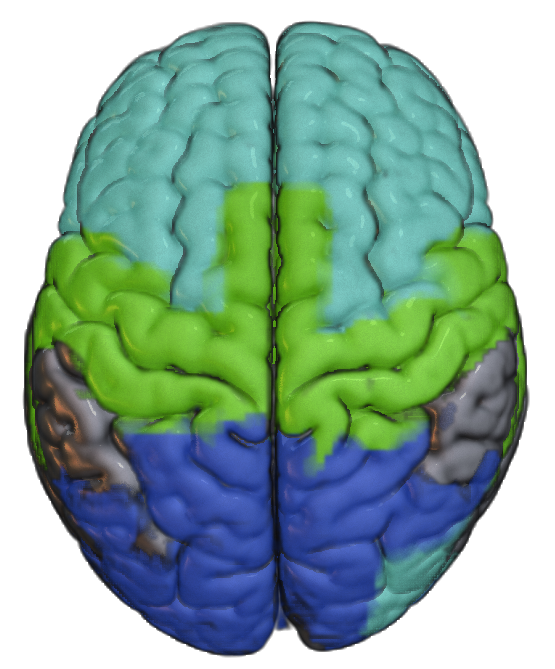

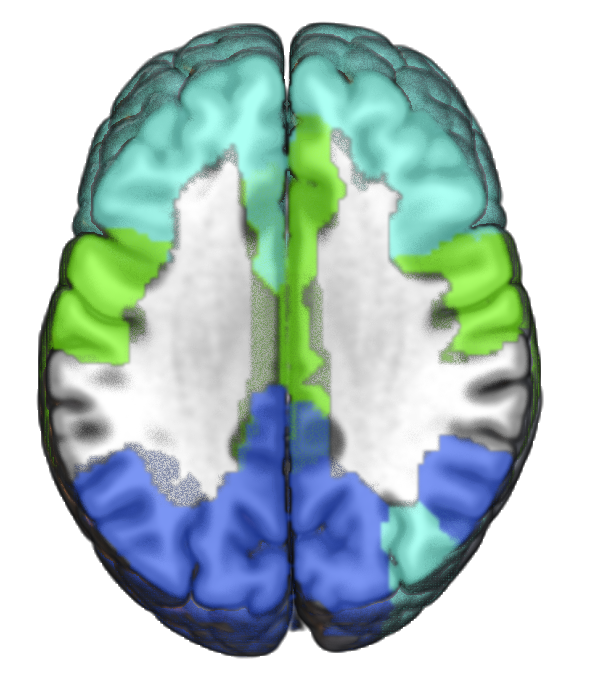

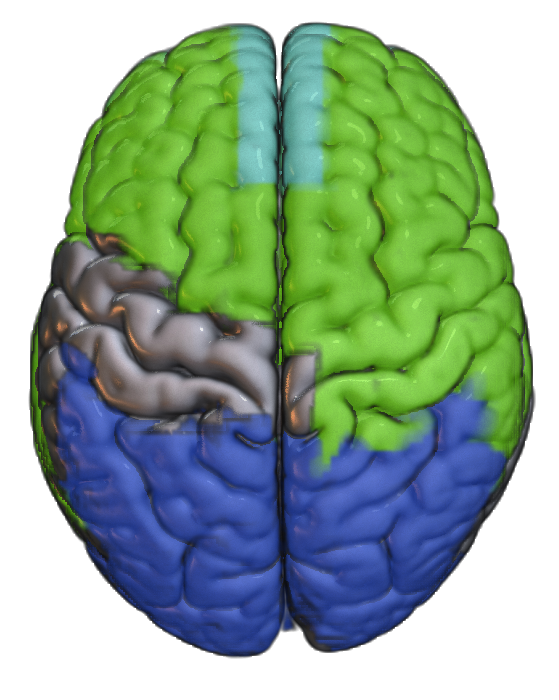

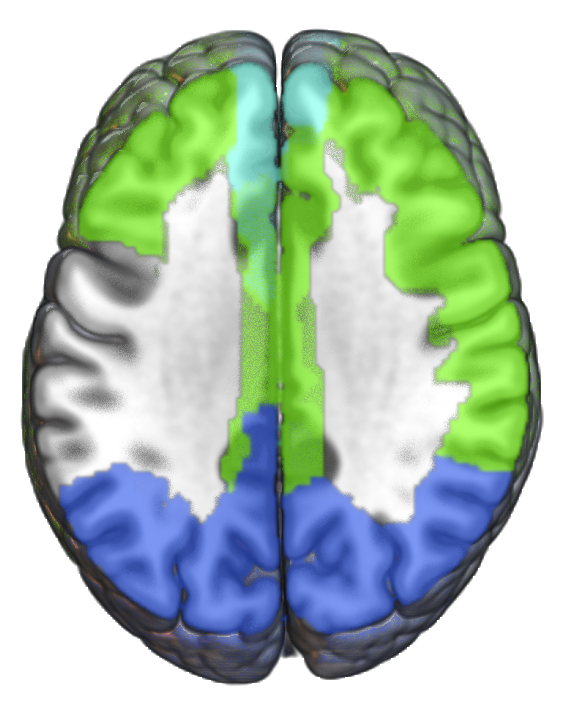

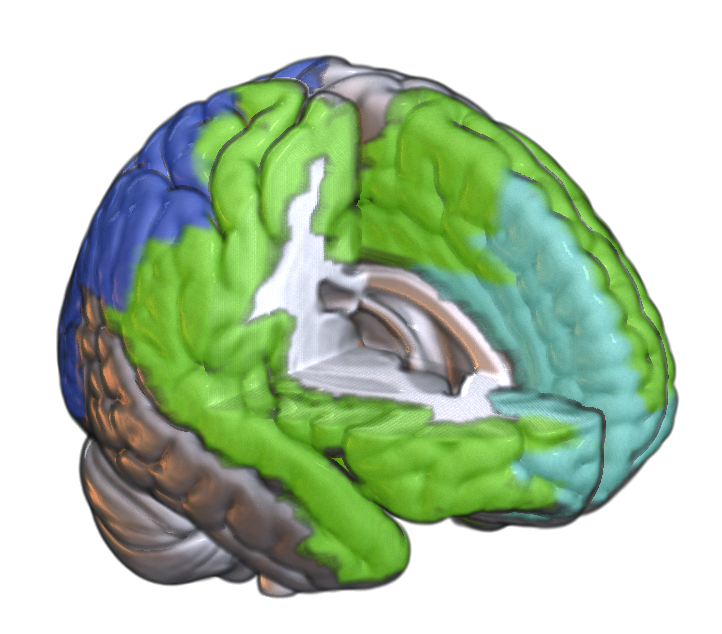

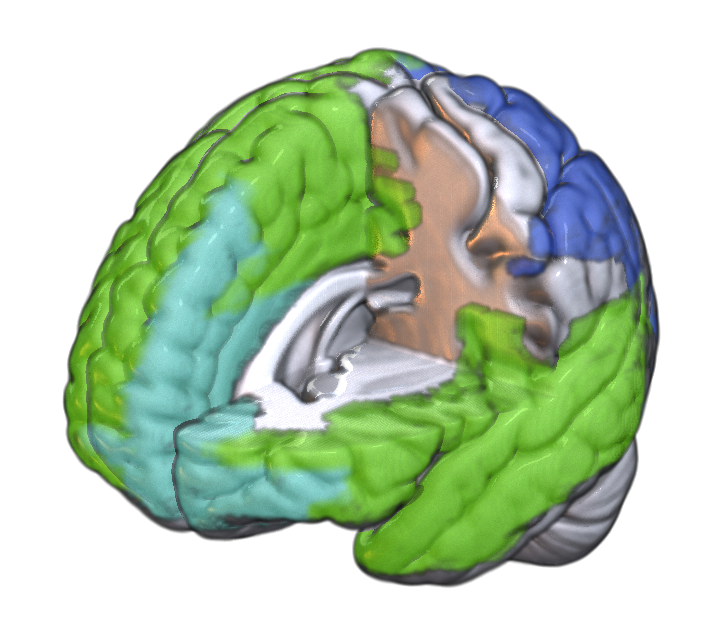

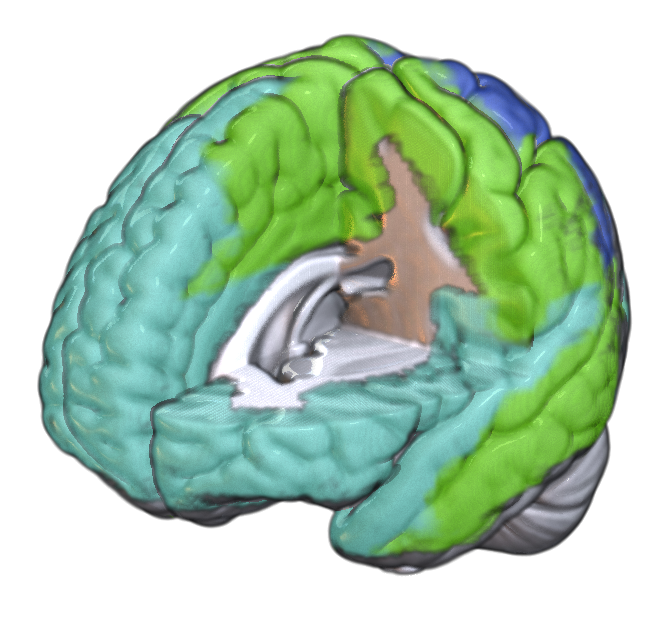

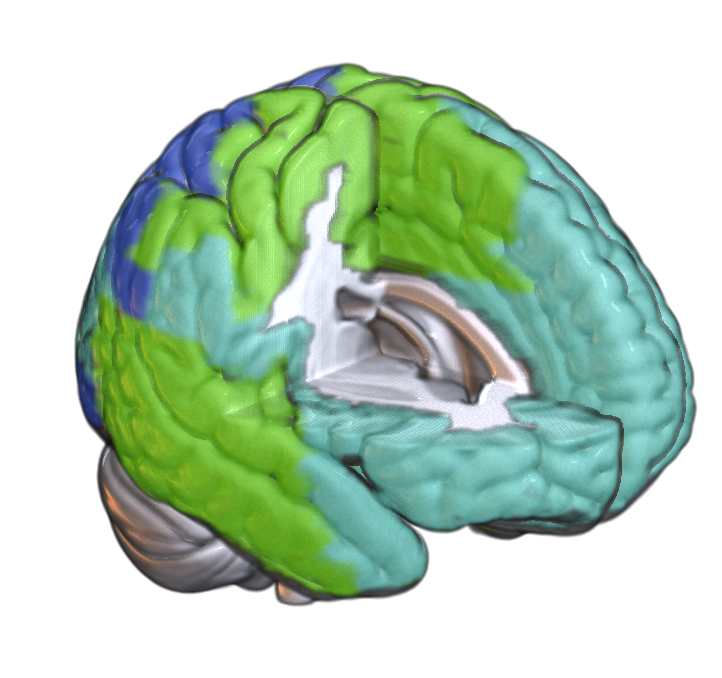

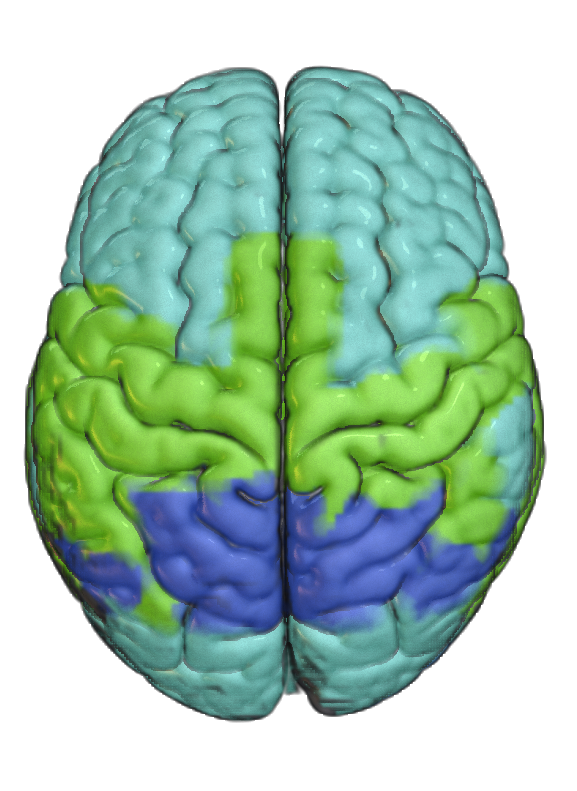

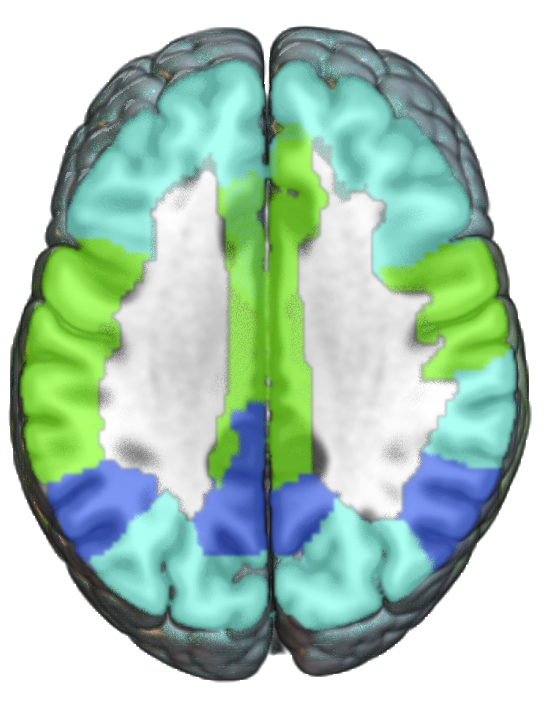

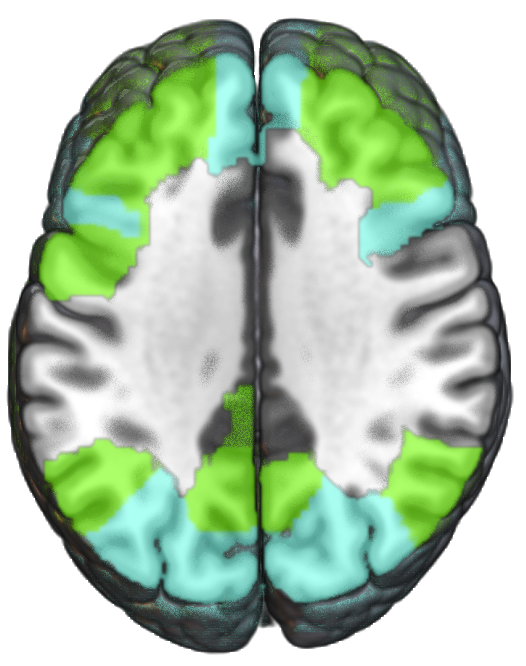

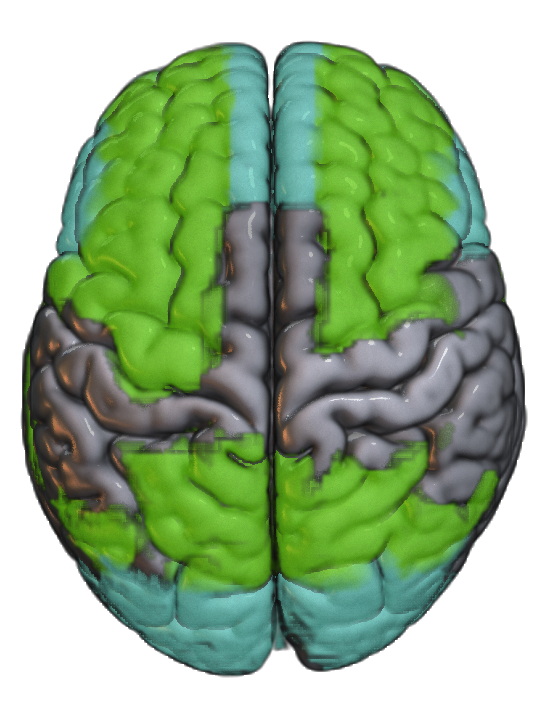

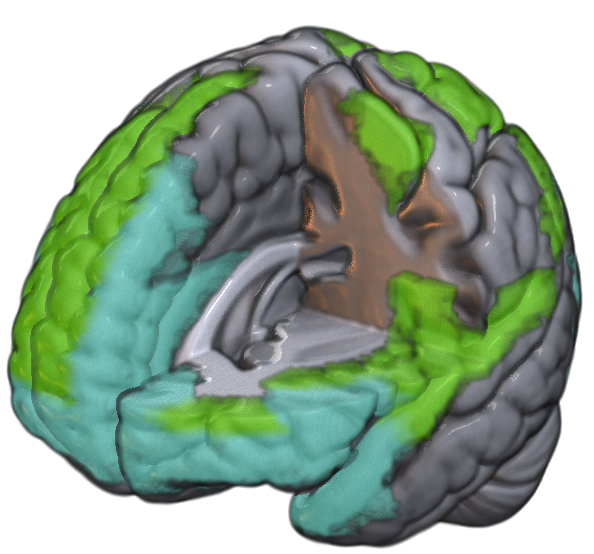

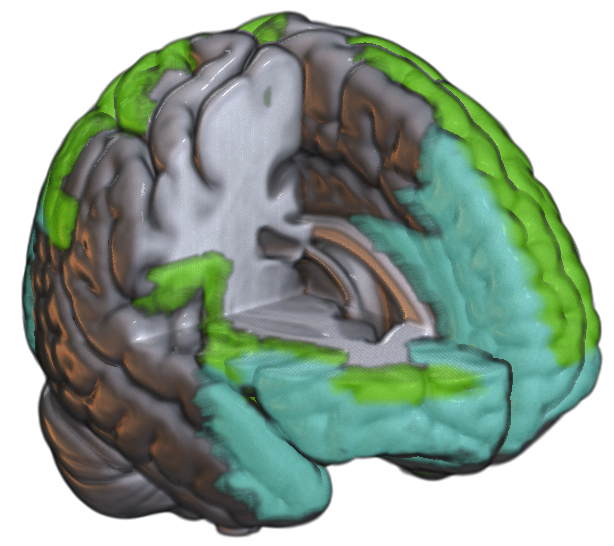


**Supplementary figure 3:** visualization of clusterization of random data compared to visualization of real electrophysiological data (theta, alpha, beta and gamma band, respectively). **A)** visualization of data from cluster 1. **B)** visualization of data from cluster 2. **C)** Dendrogram and cohesion/coherence parameters of the random data clustering. In blue, the parameters of the first cluster; in red, the parameters of the second cluster.

C)

Cut-off: 1.5

Cut-off: 1.6

**2**

**1**

**Parameters of cohesion:**

Silhouette score: 0.0078

Calinski-Harabasz score: 1.6323

Davies-Bouldin score: 3.623

RANDOM DATA

Θ

α

β

γ

B)

RANDOM DATA

A)

Θ

α

β

γ
