# Supplementary material for: Clustering Electrophysiological Predisposition to Binge Drinking: An Unsupervised Machine Learning Analysis
Source: Brain Behav. 2024 Nov 22;14(11):e70157. doi: 10.1002/brb3.70157 (PMC11583822; doi:10.1002/brb3.70157)
Supplement: Supplementary file 4 — Figure S4. Representation of different clusterization, based on specific linkage criterion, of gamma frequency band data, visualizing the random or plausible patterns generated by the different criterion. (A) Simple and geometric criterions of linkage; this type of criteria generate random‐like clusterization. (B) Complex criteria, like Ward's minimum variance (the one used on the study). (C) Weighted linkage criterion. While producing Ward‐like clusterization, generates a smoothing of the data, reducing the information given by the clusterization. [file BRB3-14-e70157-s005.docx]

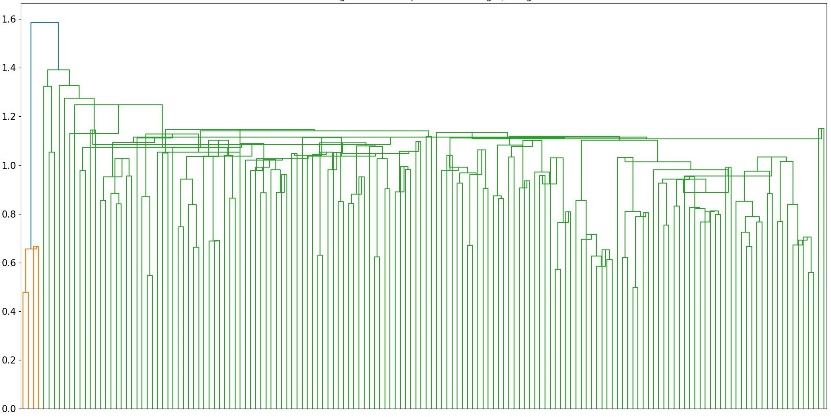

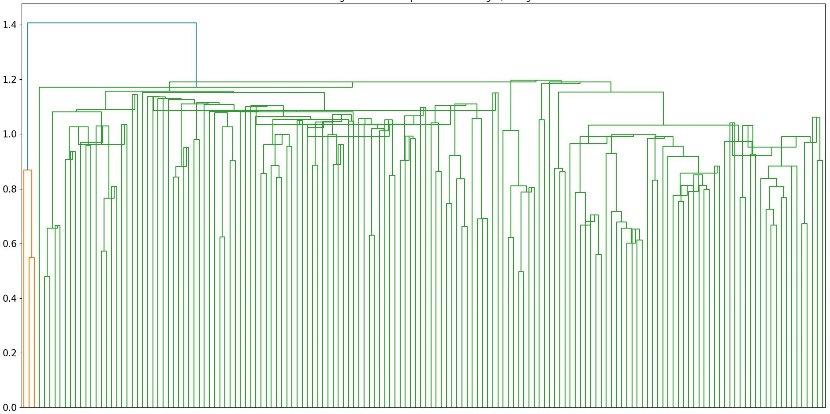

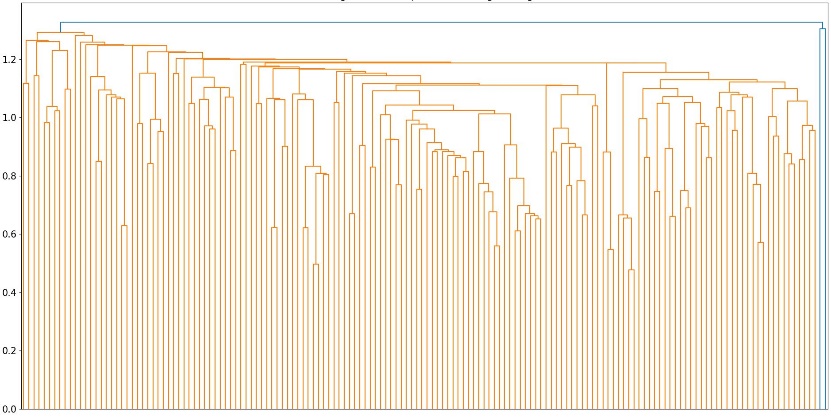

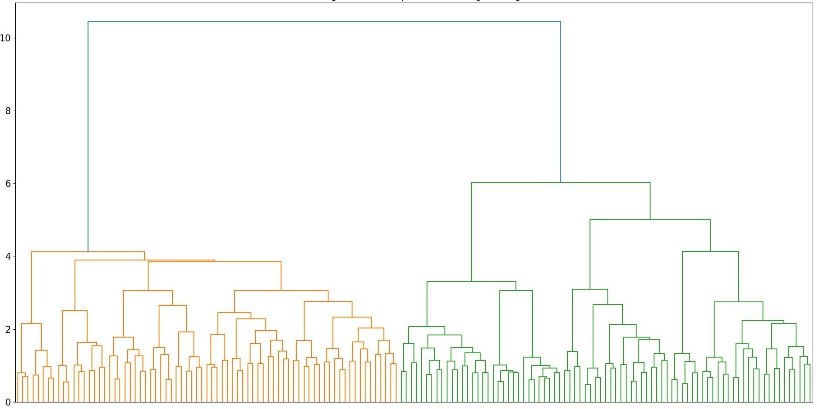

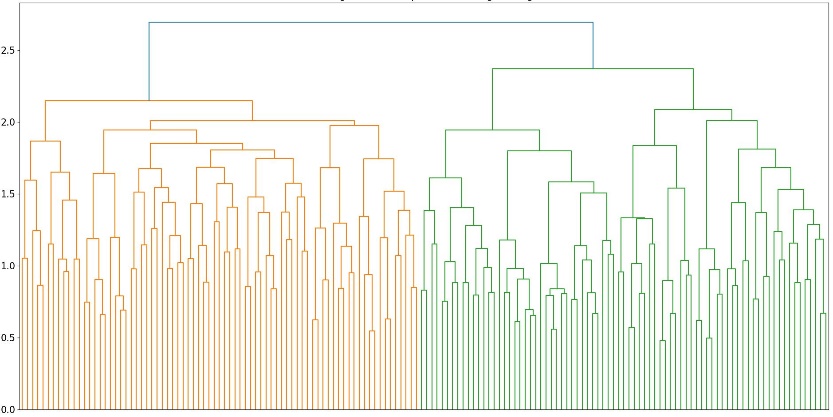

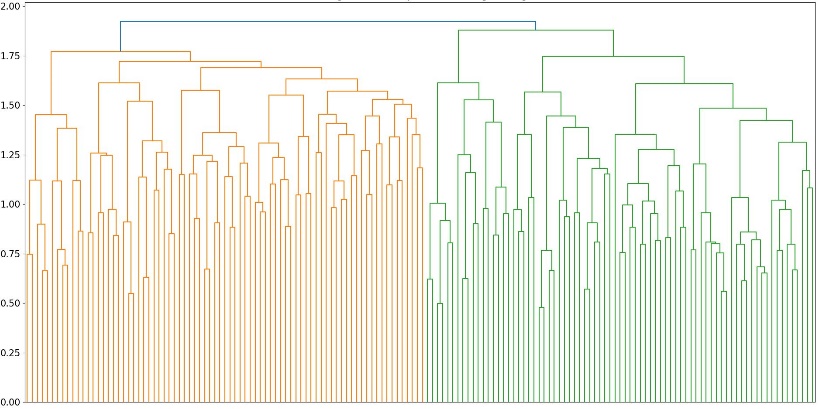

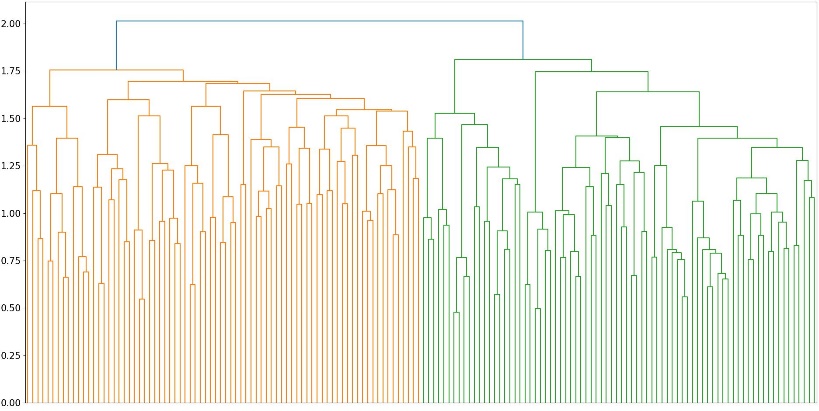


**Supplementary figure 3:** representation of different clusterization, based on specific linkage criterion, of gamma frequency band data, visualizing the random or plausible patterns generated by the different criterion. **A)** simple and geometric criterions of linkage; this type of criteria generate random-like clusterization. **B)** complex criteria, like Ward’s minimum variance (the one used on the study). **C)** Weighted linkage criterion. While producing Ward-like clusterization, generates a smoothing of the data, reducing the information given by the clusterization.

Centroid linkage

Single linkage

A)

Median linkage

Complete linkage

Ward linkage

Weighted linkage

Average linkage

C)

B)
