# Supplementary material for: Clustering Electrophysiological Predisposition to Binge Drinking: An Unsupervised Machine Learning Analysis
Source: Brain Behav. 2024 Nov 22;14(11):e70157. doi: 10.1002/brb3.70157 (PMC11583822; doi:10.1002/brb3.70157)
Supplement: Supplementary file 5 — Table S1. Sample demographics. Number of participants belonging to each sex, with percentage relative to the total sample; number of participants with country of birth in Spain, and with country of birth other than Spain, with percentage relative to the total sample; number of participants with mother tongue Spanish and with mother tongue other than Spanish, with percentage relative to the total sample; mean age, in years (with standard deviation); mean weight, in kilograms (with standard deviation); mean height, in centimeters (with standard deviation); mean study time, in hours, self‐reported by the participant (with standard deviation); mean level of parental and maternal education (with standard deviation), from 1 to 4, where 1 represents basic levels of education, and 4 represents advanced levels of education; mean health status (with standard deviation), self‐reported by the participants, from 1 to 9, where 1 represents low levels of health, and 9 represents ideal health status; and main scores of the neuropsychological tests run throughout the study, with sensation‐seeking tests (SSS‐V), executive function tests (BDEFS‐20, BRIEF‐SR, DEX) and impulsivity tests (BIS‐11) (reported values are from the total scores of the tests, not sub‐scores of said tests). [file BRB3-14-e70157-s006.docx]

| \| **Characteristic** \| \| **N** \| **%** \| \| --- \| --- \| --- \| --- \| \| Gender \| Female \| 53 \| 51 \| \| Male \| 50 \| 49 \| \| Home Country \| Spain \| 97 \| 94 \| \| Other \| 6 \| 6 \| \| Native Language \| Spanish \| 102 \| 99 \| \| Other \| 1 \| 1 \| \| **Characteristic** \| \| **Mean (± Std. Deviation)** \| \| \| Age (years) \| \| 13.75 ± 0.64 \| \| \| Weight (Kg) \| \| 50.01 ± 8.33 \| \| \| Height (cm) \| \| 161.17 ± 10.19 \| \| \| Study Time per day (hours) \| \| 1.93 ± 0.88 \| \| \| Paternal Level of Education (1 to 4) \| \| 2.87 ± 1.28 \| \| \| Maternal Level of Education (1 to 4) \| \| 3.08 ± 1.25 \| \| \| Participant's level of health (1 to 9) \| \| 8.55 ± 0.82 \| \| \| SSS-V test (total score) \| \| 18.57 ± 6.32 \| \| \| BDEFS-20 test (total score) \| \| 37.25 ± 10 \| \| \| BRIEF-SR test (metacognition index) \| \| 64.36 ± 11.3 \| \| \| BRIEF-SR test (behavioural regulation index) \| \| 58.76 ± 10.4 \| \| \| DEX test (total score) \| \| 22.99 ± 12.89 \| \| \| BIS-11 test (total score) \| \| 58.73 ± 14.07 \| \| |
| --- | --- | --- | --- | --- | --- | --- | --- | --- | --- | --- | --- | --- | --- | --- | --- | --- | --- | --- | --- | --- | --- | --- | --- | --- | --- | --- | --- | --- | --- | --- | --- | --- | --- | --- | --- | --- | --- | --- | --- | --- | --- | --- | --- | --- | --- | --- | --- | --- | --- | --- | --- | --- | --- | --- | --- | --- | --- | --- | --- | --- | --- | --- | --- | --- | --- | --- | --- | --- | --- | --- | --- | --- | --- | --- | --- | --- | --- | --- | --- | --- | --- |

**Supplementary Table 1:** Sample Demographics. Number of participants belonging to each sex, with percentage relative to the total sample; number of participants with country of birth in Spain, and with country of birth other than Spain, with percentage relative to the total sample; number of participants with mother tongue Spanish and with mother tongue other than Spanish, with percentage relative to the total sample; mean age, in years (with standard deviation); mean weight, in kilograms (with standard deviation); mean height, in centimeters (with standard deviation); mean study time, in hours, self-reported by the participant (with standard deviation); mean level of parental and maternal education (with standard deviation), from 1 to 4, where 1 represents basic levels of education, and 4 represents advanced levels of education; mean health status (with standard deviation), self-reported by the participants, from 1 to 9, where 1 represents low levels of health, and 9 represents ideal health status; and main scores of the neuropsychological tests run throughout the study, with sensation-seeking tests (SSS-V), executive function tests (BDEFS-20, BRIEF-SR, DEX) and impulsivity tests (BIS-11) (reported values are from the total scores of the tests, not sub-scores of said tests).
