# Supplementary material for: Clustering Electrophysiological Predisposition to Binge Drinking: An Unsupervised Machine Learning Analysis
Source: Brain Behav. 2024 Nov 22;14(11):e70157. doi: 10.1002/brb3.70157 (PMC11583822; doi:10.1002/brb3.70157)
Supplement: Supplementary file 6 — Table S2. Metrics of efficiency for the unsupervised machine learning model for each frequency band. For each band, the used metrics were: within‐cluster sum of squares (WCSS), silhouette score, Calinski‐Harabasz index, and Davies‐Bouldin index. Highlighted in grey, for each frequency band and metric, is the most efficient k number of groups. [file BRB3-14-e70157-s003.docx]

|  |  | *K* | | | | | | | | |
| --- | --- | --- | --- | --- | --- | --- | --- | --- | --- | --- |
|  |  | 2 | 3 | 4 | 5 | 6 | 7 | 8 | 9 | 10 |
| Θ Band | WCSS | 154.6034 | 84.2040 | 65.1970 | 55.6886 | 46.0976 | 41.7077 | 38.1897 | 35.7678 | 32.3149 |
|  | Silhouette Score | 0.4285 | 0.2301 | 0.2533 | 0.2706 | 0.2650 | 0.2592 | 0.2473 | 0.2569 | 0.2474 |
|  | C-H Index | 63.5404 | 43.1311 | 43.0969 | 42.9378 | 38.7498 | 35.6109 | 32.7579 | 30.8730 | 28.5877 |
|  | D-B Index | 0.9268 | 1.4663 | 1.3403 | 1.2464 | 1.2672 | 1.2367 | 1.3283 | 1.2375 | 1.1912 |
| α Band | WCSS | 138.9870 | 78.6026 | 55.9237 | 46.0094 | 37.7057 | 32.5953 | 28.9822 | 26.5435 | 23.9684 |
|  | Silhouette Score | 0.4066 | 0.2209 | 0.2825 | 0.3101 | 0.2866 | 0.2863 | 0.3133 | 0.3213 | 0.3012 |
|  | C-H Index | 58.3850 | 38.7396 | 45.2584 | 48.6730 | 43.1627 | 40.3903 | 42.2453 | 40.5921 | 37.1981 |
|  | D-B Index | 0.9519 | 1.5961 | 1.3126 | 1.1159 | 1.2523 | 1.2530 | 1.1285 | 1.0980 | 1.1553 |
| β Band | WCSS | 169.4634 | 89.7520 | 61.6242 | 51.3070 | 43.8194 | 38.5015 | 33.7515 | 30.6062 | 28.3573 |
|  | Silhouette Score | 0.3957 | 0.3324 | 0.2762 | 0.2759 | 0.2583 | 0.2829 | 0.2644 | 0.2753 | 0.2580 |
|  | C-H Index | 67.1292 | 65.3946 | 53.2728 | 51.3978 | 46.2070 | 46.4659 | 43.5524 | 43.1512 | 40.0250 |
|  | D-B Index | 1.0315 | 1.1129 | 1.2008 | 1.2281 | 1.3434 | 1.2724 | 1.2664 | 1.2361 | 1.2842 |
| γ Band | WCSS | 164.7371 | 107.1025 | 89.3267 | 74.2502 | 65.9369 | 57.1435 | 50.2727 | 43.6842 | 41.5075 |
|  | Silhouette Score | 0.2963 | 0.1882 | 0.2092 | 0.2407 | 0.2621 | 0.2577 | 0.2502 | 0.2131 | 0.2236 |
|  | C-H Index | 40.8645 | 26.1696 | 26.6146 | 27.0751 | 26.7370 | 27.0819 | 26.9692 | 24.1835 | 23.9817 |
|  | D-B Index | 1.3407 | 1.8892 | 1.5853 | 1.3407 | 1.2006 | 1.1312 | 1.1404 | 1.2494 | 1.2965 |

**Supplementary Table 2:** Metrics of efficiency for the unsupervised machine learning model for each frequency band. For each band, the used metrics were: Within-Cluster Sum of Squares (**WCSS**), **Silhouette score**, **Calinski-Harabasz index**, and **Davies-Bouldin index**. Highlighted in grey, for each frequency band and metric, is the most efficient *k* number of groups.
